# Influence of Tobacco on Phthisis

**Published:** 1842-10-29

**Authors:** 


*Influence of Tobacco on Phthisis.*—M. Maurice Ruef has recently published in the Gazette Medicale the following statement on this subject:

In 1836, I published a paper on the influence of tobacco on the health of the workmen in the Royal Manufactories. I there put forth the following statement: 'Pulmonary consumption is rare among the workmen who are engaged from their youth in the manipulation of tobacco; moreover, this disease makes much less rapid progress than it does usually in those who may happen to have the germ of it already developed when they enter the workshops.' My experience during the last six years has amply confirmed the accuracy of this statement, and, thanks to the improved organization of the medical service in the Royal Manufactories, I am now able to collect materials more easily than hitherto. My only motive, at present, is to direct the attention of medical men in general to the circumstance now mentioned, and to solicit them to examine the question for themselves.

